# Early prediction of in-hospital acute brain injury following cardiac arrest using machine learning: development and external validation

**DOI:** 10.3389/fneur.2026.1887511

**Published:** 2026-09-18

**Authors:** Yanxiang Niu, Tao Xu, Jianqi Fan, Jinxiang Wang, Ziquan Liu, Guowu Xu, Haojun Fan

**Affiliations:** 1 Institute of Disaster and Emergency Medicine, Tianjin University, Tianjin, China; 2 Tianjin University School of Medicine, Tianjin University, Tianjin, China; 3 College of Intelligence and Computing, Tianjin University, Tianjin, China; 4 Department of Emergency Medicine, Tianjin Medical University General Hospital, Tianjin, China

**Keywords:** brain injury, cardiac arrest, machine learning, prediction model, SHAP analysis

## Abstract

**Background:**

Neurological injury is common after cardiac arrest (CA), but early and accurate assessment remains challenging. Existing evaluation methods often lack sensitivity and specificity. This study aims to develop and validate an interpretable machine learning model for early and reliable assessment of brain injury and its prognostic relevance in ICU patients.

**Methods:**

In this study, we analyzed 1,419 CA patients from the MIMIC-IV database, randomly split into training and validation cohorts. Six machine learning (ML) algorithms-logistic regression (LR), XGBoost, artificial neural network (ANN), random forest (RF), support vector machine (SVM), and k-nearest neighbors (KNN) were developed and evaluated. The top-performing model was interpreted using SHapley Additive exPlanations (SHAP), identifying eight key predictors of brain injury. Finally, clinical data from cardiac arrest patients at our hospital were collected for external validation to assess the model’s generalizability and practical utility in an ICU setting.

**Results:**

Among the six machine learning models, the Random Forest model achieved the highest numerical performance in both the internal test and external validation cohorts. In the internal test cohort, Random Forest achieved an ACC of 88.7%, sensitivity of 80.0%, specificity of 90.6%, F1 score of 0.714, NPV of 95.5%, PPV of 64.5%, and AUC of 0.901. In the external validation cohort, Random Forest achieved an ACC of 88.8%, sensitivity of 77.8%, specificity of 90.8%, F1 score of 0.677, NPV of 95.8%, PPV of 60.0%, and AUC of 0.889. SHAP analysis identified eight influential predictors: age, hemoglobin, OASIS, temperature, blood urea nitrogen, white blood cell count, non-invasive diastolic pressure, and non-invasive systolic pressure.

**Conclusion:**

Machine learning offers strong potential for early prediction of acute brain injury in in-hospital cardiac arrest patients. Our interpretable model addresses the “black box” concern by providing transparent insights into key predictive features.

## 1 Introduction

Cardiac arrest (CA) remains a major global health burden, with substantial variation in incidence and survival across healthcare systems (1). Among patients who achieve return of spontaneous circulation, global cerebral ischemia followed by reperfusion can trigger a complex cascade involving excitotoxicity, oxidative stress, neuroinflammation, microvascular dysfunction, and neuronal death (2, 3). Post-cardiac arrest brain injury is a major cause of prolonged coma, in-hospital mortality, and long-term disability among resuscitated patients (4). Early identification of patients at increased risk of brain injury is therefore important for neurological monitoring, diagnostic evaluation, and individualized post-resuscitation management.

Clinical assessment of post-cardiac arrest brain injury commonly integrates neurological examination, electroencephalography (EEG), neuroimaging, somatosensory evoked potentials, and circulating biomarkers (5–7). However, brain injury may evolve dynamically after resuscitation, and its early manifestations can be affected by sedation, temperature management, metabolic abnormalities, and hemodynamic instability (8). In addition, comprehensive multimodal assessment often requires specialized equipment, repeated examinations, and clinical expertise that may not be uniformly available across institutions. Routinely collected electronic health record data, including demographic characteristics, vital signs, laboratory measurements, treatment information, and severity-of-illness scores-may therefore provide a complementary and readily accessible source of information for early risk assessment in ICU patients after cardiac arrest.

Machine-learning methods have increasingly been investigated for post-cardiac arrest neurological assessment. Topjian et al. combined early EEG features with clinical variables in pediatric patients after cardiac arrest and reported areas under the receiver operating characteristic curve (AUROCs) of 0.90 for unfavorable neurobehavioral outcome and 0.83 for mortality (9). Kawai et al. subsequently developed an explainable transfer-learning model using head computed tomography images obtained within 3 h after resuscitation and reported a precision-recall area under the curve (PR-AUC) of 0.73 for predicting poor neurological outcome at 1 month (10). A systematic review further showed that EEG-based machine-learning models achieved AUROCs ranging from 0.70 to 0.97, although the included studies varied considerably in model design, EEG features, and outcome definitions (11). Machine-learning models based on routinely collected clinical data have also been developed to predict in-hospital mortality among ICU patients after cardiac arrest (12, 13). More recently, Rawshani et al. developed an interpretable XGBoost model using information available at hospital admission from 55,615 patients with out-of-hospital cardiac arrest. Their model achieved an AUROC of 0.964 for predicting neurological outcome and demonstrated good calibration in an independent test set (14). Despite these advances, important gaps remain. First, models based on EEG or neuroimaging may have limited accessibility. Second, models using routine ICU data have primarily predicted mortality or functional neurological outcomes rather than in-hospital acute brain injury. Third, differences in predictor windows and outcome definitions, together with limited external validation and interpretability, may restrict model generalizability and clinical application. Therefore, an interpretable, externally validated model using routinely available ICU data is needed to predict subsequent in-hospital acute brain injury.

Accordingly, this study aimed to develop and externally validate a machine-learning model using routinely available clinical data from the first 24 h of ICU admission to predict subsequent in-hospital acute brain injury in patients with IHCA. Patients with acute brain injury documented within the 24-h predictor window were excluded, and outcomes were assessed thereafter until hospital discharge. Six machine-learning algorithms were compared, and the selected model was evaluated in an independent hospital cohort. SHapley Additive exPlanations (SHAP) were used to provide global and patient-level interpretations of model predictions without implying causality (15).

## 2 Materials and methods

This section primarily provides an overview of the data sources, criteria for inclusion and exclusion, key clinical data selected, sampling techniques employed, and classification methodologies utilized in the current study.

### 2.1 Data description

This retrospective cohort study primarily used data from the Medical Information Mart for Intensive Care IV database (MIMIC-IV, version 2.2). MIMIC-IV contains deidentified clinical data from patients admitted to the intensive care unit (ICU) or emergency department at Beth Israel Deaconess Medical Center (BIDMC), including demographic characteristics, laboratory results, and bedside monitoring parameters (16). One author (Xu; certification ID: 12556712) obtained authorized access to the database after completing the required training. As MIMIC-IV contains deidentified data, individual informed consent was not required for the present analysis. All data were accessed and analyzed in accordance with the applicable data-use requirements.

An independent external validation cohort was retrospectively collected from Tianjin Medical University General Hospital and comprised 179 critically ill patients admitted between January 2022 and March 2024. The study protocol was reviewed and approved by the Ethics Committee of Tianjin Medical University General Hospital (IRB2021-YX-240-01). Given the retrospective nature of the study, the requirement for informed consent was waived by the Ethics Committee.

### 2.2 Cardiac arrest cohort-inclusion and exclusion criteria

#### 2.2.1 Patients

Data were extracted from MIMIC-IV using Structured Query Language queries executed in PostgreSQL version 15.3 (PostgreSQL Global Development Group). Patients with in-hospital cardiac arrest were identified using the relevant International Classification of Diseases, Ninth and Tenth Revisions codes: 427.5, I46, I46.2, I46.8, and I46.9. For patients with multiple hospital or ICU admissions, only the earliest eligible admission was included.

The exclusion criteria were as follows: (1) age younger than 18 years; (2) a documented history of cardiac arrest before the index event; (3) pre-existing neurological disorders; (4) traumatic brain injury; (5) an ICU length of stay shorter than 24 h; (6) missing essential clinical information; and (7) acute brain injury documented before or during the first 24 h after ICU admission. The final prediction cohort therefore included only patients who remained free of documented acute brain injury throughout the 24-h predictor window.

The primary outcome was newly documented acute brain injury occurring after the first 24 h of ICU admission and before hospital discharge. Patients with a qualifying diagnosis first documented after the 24-h predictor window were assigned to the acute brain injury group, whereas those without a qualifying diagnosis during the remaining hospitalization were assigned to the non-brain injury group. Acute brain injury was identified using the prespecified ICD-9 and ICD-10 codes 348.1, G93.1, and I61.0-I61.9. These codes represent anoxic brain damage and nontraumatic intracerebral hemorrhage. The temporal relationship between predictor collection and outcome ascertainment is illustrated in Figure 1.

**Figure 1:**
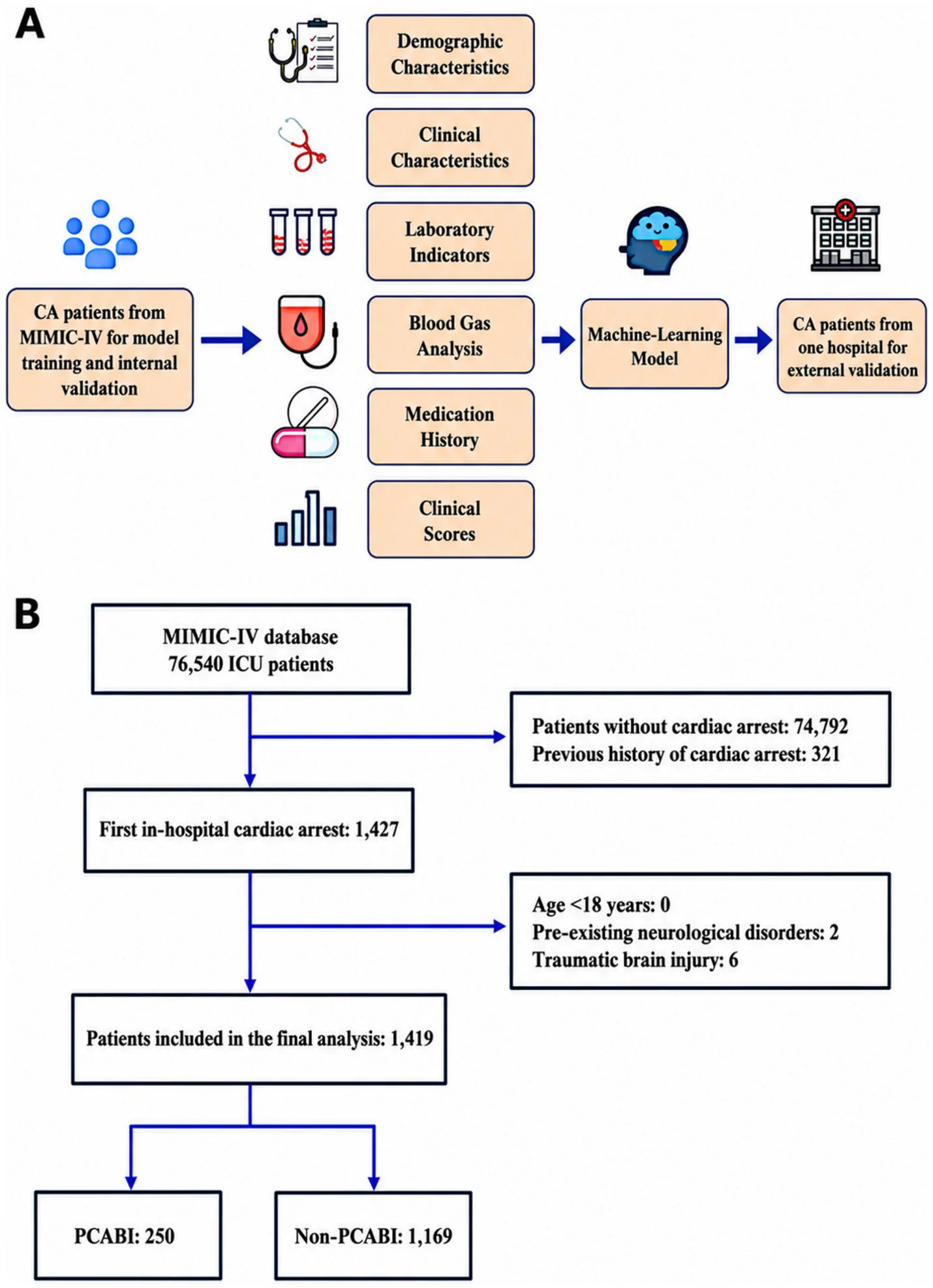
Study workflow and patient selection. **(A)** Workflow for model development, internal validation, and external validation. **(B)** Patient selection from the MIMIC-IV database. Predictors were collected within the first 24 h of ICU admission. CA, cardiac arrest; PCABI, post-cardiac-arrest brain injury.

#### 2.2.2 Data extract

In addition to clinical outcome variables (patient mortality, discharge status, and duration of ICU stay), the following variables were also extracted: demographic information (age, gender, race, height, weight, marital status), comorbidities (Coronary Heart Disease, Heart Failure, Acute Renal Failure, Hypertension, Diabetes Mellitus, Chronic Kidney Disease), and critical vital signs recorded on the initial day following admission to the Intensive Care Unit (ICU) [weight, heart rate (HR), Non-Invasive Systolic Blood Pressure (NISBP), Arterial Systolic Pressure (ASP), Arterial Diastolic Pressure (ADP), Non-Invasive Diastolic Pressure (NIDP), Respiration Rate (RR), Oxygen Saturation (SpO2)]. Additionally, laboratory test results upon ICU admission were included, comprising White Blood Cell count (WBC), Hemoglobin, Platelet count (PLT), Creatinine (Cr), Glucose, Hematocrit, Sodium (Na+), Potassium (K+), Chloride (Cl-), pH, Partial Pressure of Carbon Dioxide (pCO2), Partial Pressure of Oxygen (pO2), Blood Gas Lactic Acid (BGLA), Total Carbon Dioxide Content (TCO2), Anion Gap (AG). The evaluation also incorporated scoring systems utilized on the first day post-ICU admission, including the Sequential Organ Failure Assessment (SOFA), Acute Physiology Score (APS) II, Systemic Inflammatory Response Syndrome (SIRS), Simplified Acute Physiology Score II (SAPS II), and Oxford Acute Severity of Illness Score (OASIS II), alongside the Glasgow Coma Scale (GCS) assessments [Eye Opening, Motor Response, Verbal Response, Total (GCS Total)]. Treatment modalities such as Renal Replacement Therapy, Mechanical Ventilation, administration of Vasoactive Drugs, Antibiotics, and β-Blockers were also documented.

All predictor variables, including vital signs, laboratory results, clinical scores, and treatment variables, were restricted to records obtained during the first 24 h after ICU admission. For variables with repeated measurements, only records with a time offset from 0 to less than 1,440 min relative to ICU admission were included. No clinical information documented after the first 24 h was used as a predictor.

### 2.3 Data preprocessing

The MIMIC-IV cohort was divided into training and internal test sets before any data-dependent preprocessing was performed. Physiologically implausible measurements were identified using prespecified validity ranges and converted to missing values. Missing continuous variables were imputed using the median, whereas missing categorical variables were imputed using the most frequent category. Categorical variables were subsequently one-hot encoded. Continuous variables were standardized for LR, KNN, SVM, and ANN, but not for the tree-based RF and XGBoost models. All preprocessing parameters were estimated exclusively from the training data and applied without refitting to the internal test and external validation cohorts.

#### 2.3.1 Variable selection

A total of 52 candidate predictors were initially selected based on previous studies, clinical relevance, and their availability during the first 24 h after ICU admission (17). Variables with more than 50% missing data, including the Glasgow Coma Scale and its component scores, were excluded before model development. Consequently, GCS variables were not included in the final models or SHAP analysis. The remaining variables were retained for subsequent preprocessing and model development.

#### 2.3.2 Missing-data handling

Missingness was assessed separately for each candidate variable in the development and external validation cohorts. After variables exceeding the 50% missingness threshold were excluded, missing continuous values were imputed using medians estimated from the training data. Missing categorical values were imputed using the most frequent category in the training data.

During five-fold cross-validation, imputation parameters were estimated independently within each training fold and applied without refitting to the corresponding validation fold. After model selection, preprocessing parameters fitted using the complete training set were applied unchanged to the internal test and external validation cohorts. Physiologically implausible measurements were converted to missing values before imputation using the same prespecified validity ranges in all cohorts. Variable-specific missingness, validity ranges, inclusion status, and imputation methods are reported in Supplementary Method S1 and Supplementary Table S1.

#### 2.3.3 Class imbalance

The outcome prevalence in the development cohort was 17.6%, indicating class imbalance. SMOTE was therefore used to balance the outcome classes (18, 19). During cross-validation, SMOTE with five nearest neighbors was applied only to the training portion of each fold after preprocessing. It was not applied to the validation folds, internal test set, or external validation cohort. This procedure prevented synthetic observations from entering the datasets used for model evaluation.

### 2.4 Classification models

Six supervised machine-learning algorithms were developed and compared: LR, KNN, RF, SVM, ANN, and XGBoost (20–25). These algorithms represent linear, distance-based, kernel-based, neural-network, and tree-based approaches. Their performance was systematically compared to identify the most suitable model for predicting subsequent in-hospital acute brain injury following IHCA.

### 2.5 Model training and evaluation

The MIMIC-IV cohort was stratified by outcome and divided into training and internal test sets at an 8:2 ratio. Hyperparameters were optimized in the training set using GridSearchCV with stratified five-fold cross-validation and ROC AUC as the selection criterion. Imputation, encoding, scaling, and SMOTE were performed within each training fold to prevent information leakage.

The optimized models were refitted using the complete training set and evaluated on the held-out internal test set. The selected model was then assessed in the external cohort without retraining. Performance was evaluated using ROC AUC, accuracy, sensitivity, specificity, F1 score, and Brier score. All metrics were reported with bootstrapped 95% confidence intervals, and calibration was assessed using calibration plots, calibration intercept, and calibration slope.

### 2.6 Model explanation

SHapley Additive exPlanations (SHAP) were used to interpret the final selected model (26). Because RF demonstrated the best overall performance in the internal evaluation, SHAP analysis was applied to the fitted RF model. Global model interpretation was performed by ranking predictors according to their mean absolute SHAP values and examining SHAP summary and dependence plots. Patient-level explanations were generated to show the contribution of individual predictors to selected predictions. Positive SHAP values indicated contributions toward a higher predicted probability of acute brain injury, whereas negative values indicated contributions toward a lower predicted probability.

### 2.7 Statistic analysis

Data analysis was conducted using Python version 3.6.5 and IBM SPSS Statistics version 23.0. Continuous variables following a normal distribution were presented as mean ± standard deviation (x̅ ± s). The homogeneity of variances was tested using Levene’s test. For comparisons between groups, an independent samples t-test was utilized if variances were equal, whereas Welch’s t-test (t’) was applied if variances were unequal. Non-normally distributed continuous variables were reported as median (interquartile range) [M (QL, QU)], with comparisons between groups made using the Mann–Whitney U test. Categorical variables were displayed as counts and percentages [*n* (%)], and comparisons between groups were carried out using the Pearson chi-squared test.

## 3 Results

### 3.1 Baseline characteristics

After applying the inclusion and exclusion criteria shown in Figure 1, a total of 1,419 patients with cardiac arrest were included in the MIMIC-IV cohort. Among them, 250 patients developed newly documented post-cardiac-arrest brain injury (PCABI) after the first 24 h of ICU admission, whereas 1,169 patients did not develop PCABI before hospital discharge. Baseline characteristics of patients with and without PCABI are summarized in Table 1.

**Table 1:** Comparison of baseline data of ICU patients in the MIMIC-IV database.

Variable	PCABI (*n* = 250)	Non-PCABI (*n* = 1, 169)	c2/Z/t/t’ value	*p*-value
Age^a^	61.00 (45.75, 72.00)	70.00 (59.00, 82.00)	7.786	<0.001
Gender^b^	163 (65.20)	679 (58.10)	4.323	0.038
Weight (kg)^a^	80.00 (68.00, 95.40)	80.00 (67.50, 95.28)	0.494	0.622
Vital signs^a^				
Heart rate (times/min)	88.00 (76.00, 109.00)	89.00 (74.00, 105.00)	0.587	0.558
Non-invasive systolic pressure (mmHg)	125.50 (105.75, 146.00)	119.00 (101.00, 138.00)	2.864	0.004
Non-invasive diastolic pressure (mmHg)	75.00 (62.00, 91.00)	67.00 (55.00, 80.00)	5.844	<0.001
Non-invasive mean pressure (mmHg)	88.00 (73.00, 103.25)	79.00 (67.86, 93.00)	5.338	<0.001
Respiration Rate (times/min)	20.00 (16.00, 25.00)	19.00 (16.00, 24.00)	2.752	0.006
SPO2 (%)	99.00 (94.00, 100.00)	98.00 (95.00, 100.00)	1.068	0.285
Temperature (°C)	36.78 (35.61, 37.28)	36.61 (36.12, 37.00)	1.263	0.207
Laboratory test				
White blood cells (×109/L)^a^	14.65 (9.95, 20.83)	11.40 (8.10, 16.40)	5.796	<0.001
Red blood cells (×1, 012/L)^c^	4.13 ± 0.91	3.80 ± 0.84	5.633^d^	<0.001
Platelets (×109/L)^a^	210.50 (156.50, 279.50)	196.00 (144.00, 259.00)	2.535	0.011
Hemoglobin (g/dL)^c^	12.44 ± 2.79	11.36 ± 2.45	5.654^e^	<0.001
Red blood cell distribution width (%)^a^	14.20 (13.30, 15.30)	14.50 (13.50, 16.00)	3.169	0.002
Sodium (mmol/L)^a^	140.00 (137.00, 143.00)	139.00 (136.00, 141.00)	3.790	<0.001
Potassium (mmol/L)^a^	4.00 (3.60, 4.50)	4.20 (3.80, 4.70)	4.362	<0.001
Chlorine (mmol/L)^a^	105.00 (101.00, 109.00)	104.00 (100.00, 107.00)	3.435	0.001
Magnesium (mg/dL)^a^	2.00 (1.80, 2.30)	2.00 (1.80, 2.30)	1.182	0.237
Blood sugar (mg/dL)^a^	171.50 (125.50, 258.75)	148.00 (110.00, 212.50)	4.185	<0.001
pH ^a^	7.28 (7.19, 7.38)	7.33 (7.21, 7.40)	3.172	0.002
PaCO2 (mmHg)^a^	41.00 (35.00, 52.00)	43.00 (36.00, 52.00)	1.040	0.298
Lactic acid (mmol/L)^a^	3.20 (1.70, 6.10)	2.40 (1.50, 4.60)	3.825	<0.001
Anion gap (mmol/L)^a^	17.00 (14.00, 21.00)	16.00 (13.00, 20.00)	3.529	<0.001
Total CO2 (mmol/L)^a^	21.00 (18.00, 24.98)	23.00 (19.00, 26.62)	4.731	<0.001
Urea nitrogen (mg/dL)^a^	21.50 (14.00, 31.00)	23.00 (16.00, 40.00)	3.235	0.001
Creatinine (mg/dL)^a^	1.20 (0.90, 1.70)	1.20 (0.80, 1.90)	0.386	0.699
Critical care scoring system^a^				
SOFA	11.00 (8.00, 13.00)	8.00 (4.00, 11.00)	7.665	<0.001
APACHE III	87.00 (64.00, 105.00)	62.00 (42.00, 87.50)	9.051	<0.001
SIRS	3.00 (3.00, 4.00)	3.00 (2.00, 4.00)	2.491	0.013
SAPS II	49.00 (38.00, 62.00)	44.00 (34.00, 57.00)	3.483	<0.001
OASIS	44.00 (38.00, 50.00)	38.00 (31.00, 46.00)	7.815	<0.001
Mechanical ventilation^b^	245 (98.00)	1, 013 (86.70)	26.352	<0.001

a Represents the median (P25, P75).

b Represents a percentage (%).

c Represents the mean ± standard deviation.

^d^Represents the t-value for homogeneous variances.

^e^Represents the t-value for heterogeneous variances.

Patients with PCABI were younger than those without PCABI, with a median age of 61 years versus 70 years, respectively (*p* < 0.001). The proportion of male patients was also higher in the PCABI group than in the non-PCABI group (65.2% vs. 58.1%, *p* = 0.038). Regarding vital signs, patients with PCABI had higher median non-invasive systolic blood pressure (125.50 vs. 119.00 mmHg, *p* = 0.004), non-invasive diastolic blood pressure (75.00 vs. 67.00 mmHg, *p* < 0.001), and non-invasive mean arterial pressure (88.00 vs. 79.00 mmHg, *p* < 0.001). Respiratory rate was also slightly higher in the PCABI group than in the non-PCABI group (20 vs. 19 breaths/min, *p* = 0.006).

For laboratory variables, patients with PCABI had higher white blood cell counts (14.65 vs. 11.40 × 10^9^/L, *p* < 0.001), red blood cell counts (4.13 ± 0.91 vs. 3.80 ± 0.84 × 10^12^/L, *p* < 0.001), and hemoglobin levels (12.44 ± 2.79 vs. 11.36 ± 2.45 g/dL, *p* < 0.001). Sodium, potassium, anion gap, and lactate also differed significantly between the two groups. In addition, patients with PCABI had higher SOFA, APACHE III, SAPS II, and OASIS scores than those without PCABI, indicating greater overall illness severity. Mechanical ventilation was more common in the PCABI group than in the non-PCABI group (98.0% vs. 86.7%, *p* < 0.001).

The baseline characteristics, outcome prevalence, predictor window, and outcome window of the external validation cohort are provided in Supplementary Table S2.

### 3.2 Model performance comparisons

In this study, we evaluated the predictive performance of six machine learning models, including random forest (RF), XGBoost, k-nearest neighbors (KNN), artificial neural network (ANN), logistic regression (LR), and support vector machine (SVM), for predicting brain injury outcomes. Model performance was assessed in both the internal test cohort from MIMIC-IV and the external hospital-based validation cohort. Accuracy, sensitivity, specificity, F1 score, negative predictive value (NPV), positive predictive value (PPV), and AUC with 95% confidence intervals are summarized in Table 2, and the corresponding ROC curves are shown in Figure 2.

**Table 2:** Performance of machine learning models (external and internal).

Cohort	Model	ACC	Sen	Spe	F1 score	NPV	PPV	AUC (95% CI)
Internal test	SVM	0.803	0.600	0.846	0.517	0.908	0.455	0.752 (0.670–0.834)
	KNN	0.835	0.700	0.863	0.598	0.931	0.522	0.806 (0.730–0.882)
	ANN	0.817	0.620	0.859	0.544	0.914	0.484	0.771 (0.691–0.851)
	LR	0.873	0.760	0.897	0.679	0.946	0.613	0.874 (0.809–0.939)
	RF	0.887	0.800	0.906	0.714	0.955	0.645	0.901 (0.843–0.959)
	XGBoost	0.856	0.720	0.885	0.637	0.937	0.571	0.832 (0.760–0.904)
External validation	SVM	0.816	0.593	0.855	0.492	0.922	0.421	0.768 (0.658–0.878)
	KNN	0.838	0.667	0.868	0.554	0.936	0.474	0.792 (0.686–0.898)
	ANN	0.827	0.630	0.862	0.515	0.929	0.447	0.789 (0.683–0.895)
	LR	0.872	0.741	0.895	0.635	0.951	0.556	0.856 (0.764–0.948)
	RF	0.888	0.778	0.908	0.677	0.958	0.600	0.889 (0.806–0.972)
	XGBoost	0.866	0.704	0.895	0.612	0.944	0.559	0.851 (0.757–0.945)

**Figure 2:**
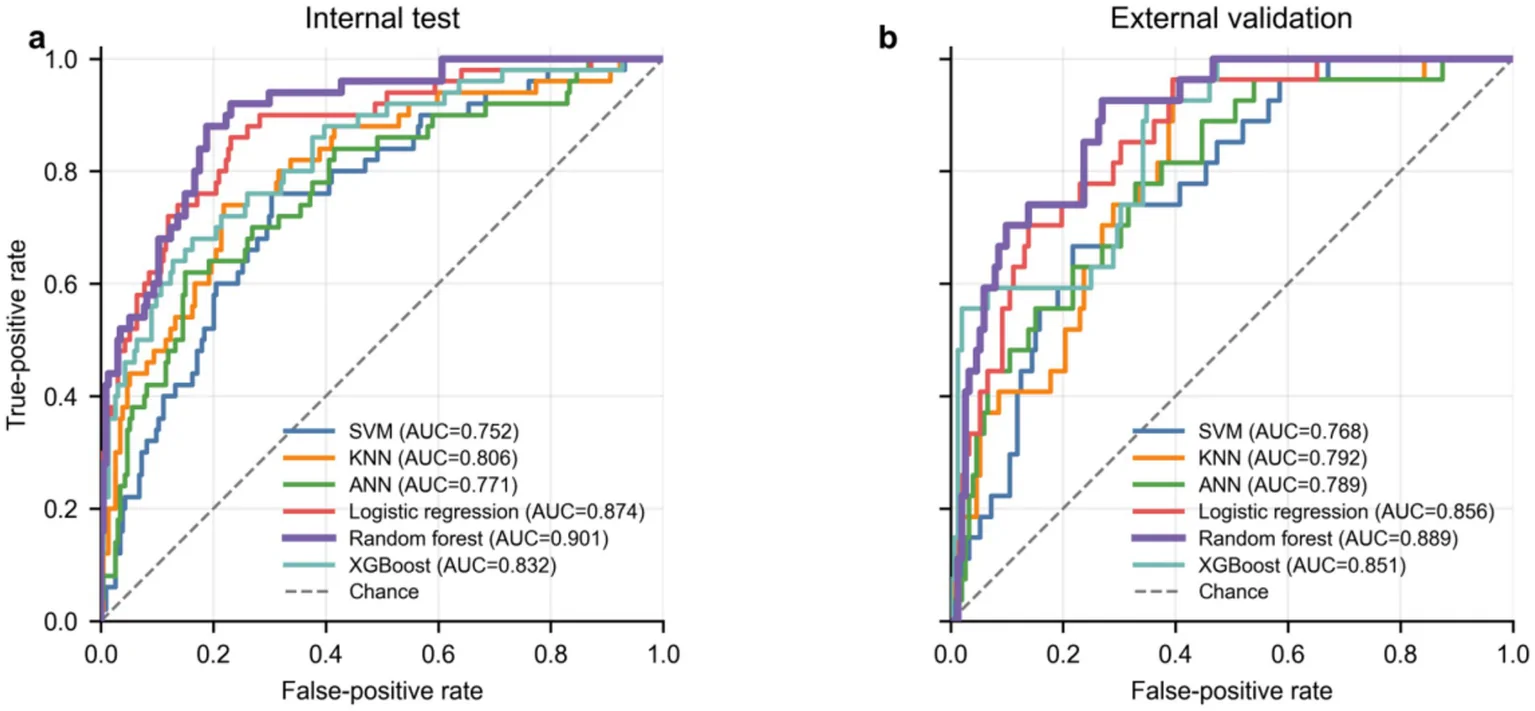
ROC curves of the six models in the internal test cohort **(a)** and external validation cohort **(b)**.

In the internal test cohort, RF achieved the highest overall discriminative performance, with an AUC of 0.901, accuracy of 0.887, sensitivity of 0.800, specificity of 0.906, and F1 score of 0.714. LR also showed strong performance, with an AUC of 0.874 and accuracy of 0.873, indicating that the predictive signal was not limited to a single non-linear model. XGBoost, KNN, ANN, and SVM demonstrated moderate discrimination, with AUCs ranging from 0.752 to 0.832.

In the external validation cohort, RF again achieved the highest numerical AUC of 0.889, with accuracy of 0.888, sensitivity of 0.778, specificity of 0.908, and F1 score of 0.677. LR and XGBoost also maintained good external performance, with AUCs of 0.856 and 0.851, respectively. Compared with the internal test cohort, the external validation performance of RF was slightly lower but remained comparable, suggesting that the model retained reasonable generalizability across datasets.

Overall, RF showed the highest numerical performance in both internal and external validation cohorts. However, LR achieved comparable discrimination with greater interpretability. Therefore, RF was considered the best-performing model numerically, while LR served as an important interpretable benchmark for evaluating the robustness of the predictive associations.

### 3.3 Application of the model

SHAP analysis was applied to the retrained Random Forest model to quantify global and individual feature contributions. Before model development, clearly implausible physiological measurements were converted to missing values. Median imputation was fitted using the training data only. Because Random Forest does not require feature standardization, SHAP values were calculated using the same imputed, unstandardized clinical values used to train the model.

Figure 3a presents the mean absolute SHAP values of eight prespecified clinical features, whereas Figure 3b shows the direction and magnitude of their contributions. Figure 3c presents the corresponding dependence patterns. Within this prespecified feature set, age had the largest mean absolute SHAP value, followed by OASIS, white blood cell count, non-invasive diastolic pressure, hemoglobin, temperature, blood urea nitrogen, and non-invasive systolic pressure.

**Figure 3:**
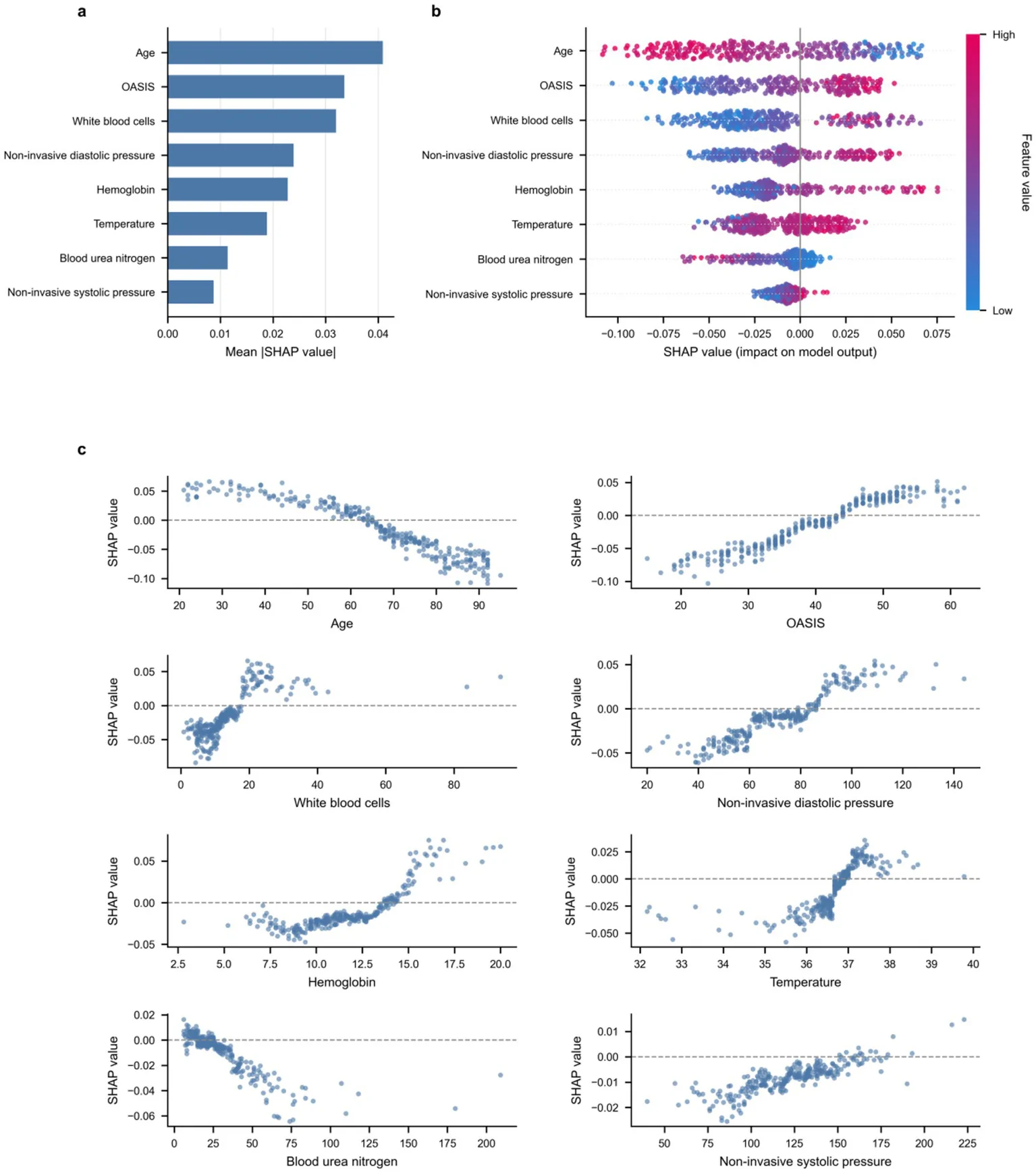
Global SHAP interpretation of the random forest model **(a)** Feature importance ranked by mean absolute SHAP values. **(b)** SHAP summary plot showing the direction and magnitude of each feature’s contribution. **(c)** SHAP dependence plots showing the relationships between feature values and model outputs. Positive SHAP values indicate contributions toward a higher predicted risk of PCABI, whereas negative values indicate contributions toward a lower predicted risk. SHAP, SHapley Additive exPlanations; PCABI, post-cardiac-arrest brain injury.

Higher OASIS values, elevated white blood cell counts, higher non-invasive diastolic pressure, and higher hemoglobin concentrations generally contributed toward higher model outputs. Temperature showed a nonlinear relationship with the model output. Older age and higher blood urea nitrogen values predominantly produced negative SHAP contributions in this cohort. Non-invasive systolic pressure had the smallest mean absolute contribution among the eight displayed features. These SHAP values describe the behavior of the fitted model and should not be interpreted as independent or causal effects.

Figure 4 presents a local SHAP explanation for an illustrative PCABI-positive prediction. The example had a predicted PCABI probability of 0.712. An age of 67 years, a white blood cell count of 17.8, non-invasive diastolic pressure of 134 mmHg, an OASIS score of 62, a hemoglobin concentration of 9.2 g/dL, and a temperature of 36.8 °C increased the model output. In contrast, a blood urea nitrogen level of 22 mg/dL and a non-invasive systolic pressure of 148 mmHg decreased the model output. The remaining features collectively had only a small negative contribution to the prediction.

**Figure 4:**
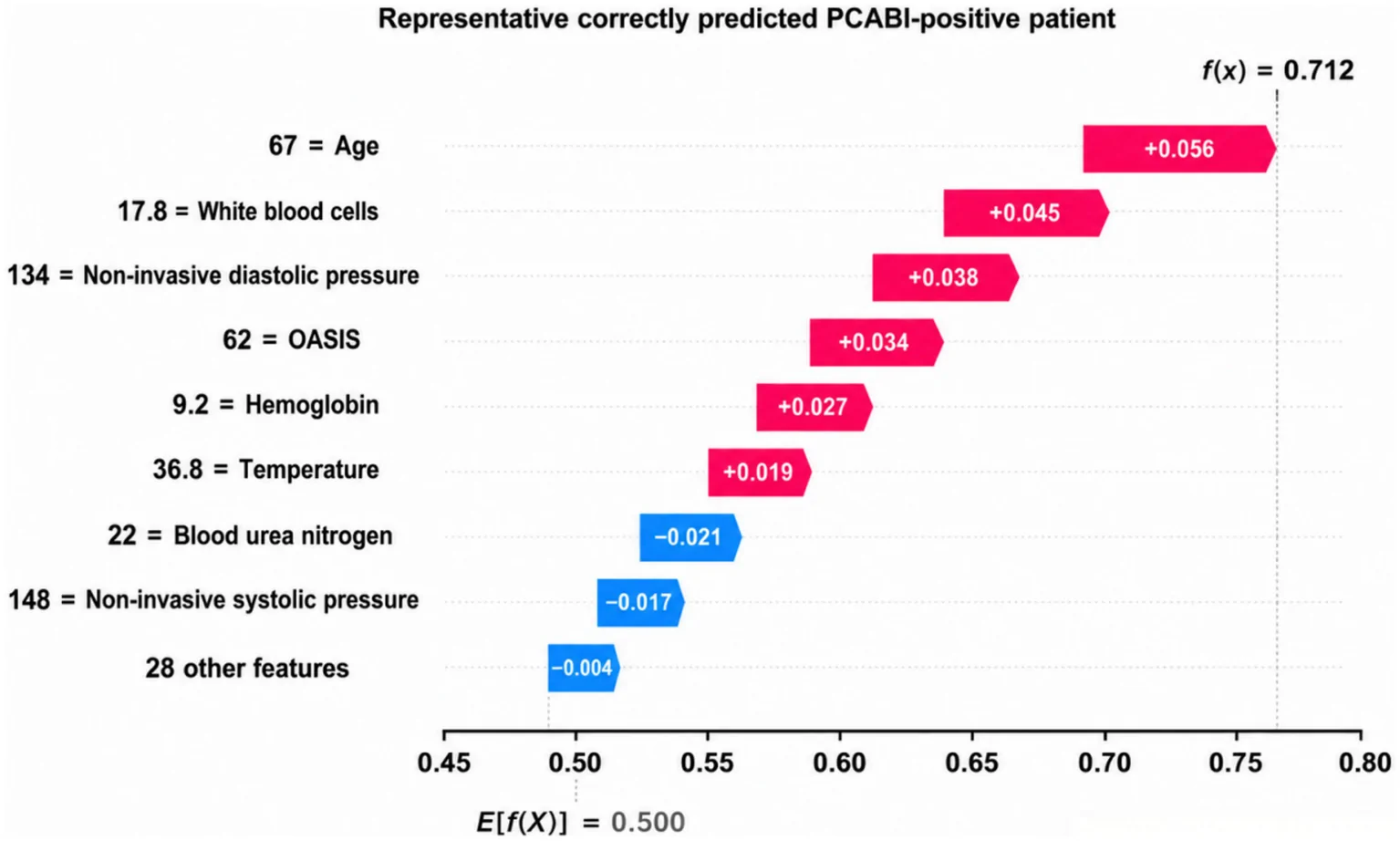
Local SHAP explanation for an individual patient. The waterfall plot shows how each feature contributed to the model output relative to the baseline value. Red and blue indicate contributions toward higher and lower predicted risks of PCABI.

## 4 Discussion

In this study, routinely collected clinical variables from the first 24 h of ICU admission were used to predict subsequent in-hospital acute brain injury after IHCA. RF achieved the best overall performance, with AUCs of 0.901 and 0.889 in the internal test and external validation cohorts, respectively. SHAP identified hemoglobin, non-invasive blood pressure, age, OASIS, temperature, white blood cell count, and blood urea nitrogen as influential contributors to model predictions. These findings describe model behavior rather than causal relationships or therapeutic targets. Although the results support further investigation of early risk stratification, additional calibration, clinical-utility assessment, and prospective multicenter validation are required before clinical implementation.

### 4.1 Model performance evaluation

Previous studies have demonstrated the potential of machine-learning approaches for clinical outcome prediction. For instance, Park et al. (27) developed a machine-learning model for predicting neurologically intact survival after out-of-hospital cardiac arrest, achieving an AUC of 0.926 with the best-performing voting classifier. Ambale-Venkatesh et al. (28) demonstrated that random survival forests improved cardiovascular event prediction compared with established risk scores in the Multi-Ethnic Study of Atherosclerosis. Rajkomar et al. (29) applied deep learning to large-scale electronic health record data and achieved AUROCs of 0.93–0.94 for in-hospital mortality prediction, outperforming traditional predictive models. Similarly, Cheng et al. (30) compared logistic regression, support vector machine, and XGBoost for neurological outcome prediction after out-of-hospital cardiac arrest and found that XGBoost achieved the highest AUC of 0.956, with a sensitivity of 0.875 and specificity of 0.904.

In the present study, RF achieved the highest overall discrimination among the evaluated models in both the internal test and external validation cohorts. SHAP analysis was used to characterize global feature importance and patient-level contributions, thereby improving transparency regarding model behavior. However, discrimination and interpretability alone do not establish clinical usefulness. Although calibration and decision-curve analyses provide additional information on agreement between predicted and observed risks and potential net benefit, these findings require cautious interpretation because external validation was limited to a relatively small single-center cohort. Accordingly, the model should be regarded as an investigational risk-stratification approach rather than a tool ready for clinical decision-making. Prospective multicenter validation and evaluation of its effects on clinical decisions and patient outcomes are required before implementation.

### 4.2 Interpretation of influential features

SHAP analysis identified hemoglobin, non-invasive diastolic pressure, age, white blood cell count, OASIS, temperature, non-invasive systolic pressure, and blood urea nitrogen as influential features in the RF model. SHAP values quantify the contributions of individual features to model predictions rather than independent associations or causal effects. Their magnitude and direction may also reflect nonlinear relationships and interactions with other variables. The following interpretations therefore provide clinical context for model behavior but should not be regarded as evidence of biological mechanisms or therapeutic targets.

Hemoglobin was an influential contributor to the RF predictions. Previous studies have associated anemia or lower hemoglobin concentrations with impaired cerebral oxygen delivery and unfavorable neurological outcomes after cardiac arrest. Wormsbecker et al. reported an association between anemia and poor neurological outcomes in patients with hypoxic–ischemic brain injury following cardiac arrest (31). The SOS-KANTO study similarly found that higher hemoglobin concentrations at hospital arrival were associated with more favorable short-term neurological outcomes (32). Ameloot et al. reported that lower hemoglobin concentrations were associated with reduced cerebral oxygen saturation and unfavorable outcomes in patients with impaired cerebral or systemic oxygenation (33). In our model, however, the direction and magnitude of the hemoglobin contribution varied according to the clinical context, as illustrated by the local SHAP explanation in Figure 4. These findings should therefore be interpreted as reflecting the behavior of the fitted model rather than an independent biological effect of hemoglobin. In particular, the SHAP results do not establish a universal hemoglobin threshold or imply that modifying hemoglobin concentrations would reduce the risk of subsequent brain injury.

Non-invasive diastolic pressure also contributed to model predictions. Previous studies have reported associations between post-arrest diastolic pressure, systemic circulation, cerebral physiology, and neurological outcomes (34–36). However, blood-pressure contributions in the present model may have been influenced by cardiac function, vasopressor administration, baseline hypertension, measurement conditions, and cerebral autoregulation. The SHAP findings therefore do not demonstrate that either higher or lower diastolic pressure directly causes brain injury, nor do they identify an optimal treatment target.

Age contributed to the RF predictions, although its direction did not consistently follow the conventional association between older age and unfavorable post-cardiac-arrest outcomes. This pattern may reflect cohort selection, comorbidity profiles, competing risks, treatment differences, or interactions with other predictors. Previous studies also indicate that the mechanisms and prognostic characteristics of post-cardiac-arrest brain injury may differ between adult and pediatric populations (37–39). Therefore, the age-related SHAP pattern should not be interpreted as evidence that younger age independently increases the biological risk of brain injury.

White blood cell count was another influential feature. Leukocytosis following cardiac arrest may reflect systemic inflammation, infection, physiological stress, or overall illness severity. Elevated leukocyte counts have previously been associated with unfavorable outcomes after severe head injury (40). More directly, Dufner et al. examined leukocyte kinetics in patients resuscitated from cardiac arrest and found that leukocyte counts were affected by targeted temperature management. Among patients receiving targeted temperature management, lower leukocyte counts on day 3 were associated with better survival and neurological outcomes (41). Nevertheless, leukocyte measurements may be influenced by infection, temperature management, medication, and the timing of blood sampling. Therefore, the SHAP contribution of white blood cell count should be interpreted as a model-based signal of systemic physiological status rather than evidence of a direct inflammatory effect on the brain.

OASIS was higher among patients with PCABI and contributed to the RF predictions. OASIS is a parsimonious measure of physiological instability developed for critically ill patients (42). Its prognostic value has also been reported in patients with acute stroke complicated by stroke-associated pneumonia and in critically ill patients with sepsis (43, 44). In the present model, the OASIS contribution may reflect overall illness severity during the initial ICU period rather than a neurological pathway specific to cardiac arrest.

Temperature contributed to model predictions despite the absence of a statistically significant univariable difference between the outcome groups. This apparent difference between the univariable analysis and SHAP results may reflect nonlinear effects or interactions with other model features. Temperature measurements may also be influenced by spontaneous fever, infection, post-resuscitation physiology, targeted temperature management, and measurement practices. Previous studies have emphasized the relationships among temperature management, systemic physiology, and neurological injury after cardiac arrest (45–47). However, the present analysis cannot distinguish spontaneous temperature abnormalities from treatment-related changes or demonstrate that modifying temperature would reduce the subsequent risk of brain injury.

Non-invasive systolic pressure was also influential in the model. Previous studies in traumatic brain injury populations have reported associations between systolic hypotension and secondary neurological injury or mortality (48–50). However, these findings cannot be directly extrapolated to patients with IHCA. Furthermore, systolic pressure recorded during the first 24 h may reflect both the patient’s circulatory condition and clinical interventions. Its SHAP contribution should therefore be interpreted as part of the model’s multivariable prediction pattern rather than evidence supporting a specific systolic-pressure target.

BUN was identified as another influential feature. BUN may reflect renal function, hydration, catabolism, circulatory status, nutritional status, and overall disease severity. An association between BUN and the type or severity of traumatic brain injury has previously been reported (51), but direct evidence in patients with cardiac arrest remains limited. Accordingly, its SHAP contribution should be regarded as a model-based signal rather than evidence that BUN is a brain-specific biomarker or causal determinant of post-cardiac-arrest brain injury.

Overall, SHAP analysis improves model transparency by showing how individual variables contributed to RF predictions. Nevertheless, SHAP importance does not establish statistical independence, causal mechanisms, therapeutic thresholds, or modifiable treatment targets. These findings should therefore be considered hypothesis-generating and require confirmation in prospective studies that account for temporal changes, clinical interventions, and relevant confounding factors.

## 5 Conclusion and future directions

This study developed and externally validated machine-learning models using routinely collected data from the first 24 h of ICU admission to predict subsequent in-hospital acute brain injury after IHCA. The RF model achieved the best overall performance, while SHAP analysis improved model transparency by characterizing global and patient-level feature contributions. These findings support the potential of early ICU data for risk stratification but do not establish causal relationships or clinical utility.

The study was limited by its retrospective design, ICD code-based outcome definition, and external validation in a relatively small single-center cohort. Moreover, neurological examination findings, EEG, neuroimaging, and neurological biomarkers were not included. Further prospective multicenter validation is therefore required, together with comprehensive assessment of calibration and clinical utility. Future studies may also evaluate whether incorporating serial neurological examinations, EEG, neuroimaging, and biomarkers provides meaningful improvement over routinely available clinical variables.

## Data Availability

The original contributions presented in the study are included in the article/Supplementary material, further inquiries can be directed to the corresponding authors.

## References

[1] Andersen LW Holmberg MJ Berg KM Donnino MW Granfeldt A. In-hospital cardiac arrest: a review. JAMA. (2019) 321:1200–10. doi: 10.1001/jama.2019.1696, 30912843 PMC6482460

[2] Perkins GD Callaway CW Haywood K Neumar RW Lilja G Rowland MJ . Brain injury after cardiac arrest. Lancet (London, England). (2021) 398:1269–78. doi: 10.1016/S0140-6736(21)00953-3, 34454687

[3] Sandroni C Cronberg T Sekhon M. Brain injury after cardiac arrest: pathophysiology, treatment, and prognosis. Intensive Care Med. (2021) 47:1393–414. doi: 10.1007/s00134-021-06548-2, 34705079 PMC8548866

[4] Lemiale V Dumas F Mongardon N Giovanetti O Charpentier J Chiche JD . Intensive care unit mortality after cardiac arrest: the relative contribution of shock and brain injury in a large cohort. Intensive Care Med. (2013) 39:1972–80. doi: 10.1007/s00134-013-3043-4, 23942856

[5] Geocadin RG Callaway CW Fink EL Golan E Greer DM Ko NU . Standards for studies of neurological prognostication in comatose survivors of cardiac arrest: a scientific statement from the American Heart Association. Circulation. (2019) 140:e517–42. doi: 10.1161/CIR.0000000000000702, 31291775

[6] Rajajee V Muehlschlegel S Wartenberg KE Alexander SA Busl KM Chou SHY . Guidelines for neuroprognostication in comatose adult survivors of cardiac arrest. Neurocrit Care. (2023) 38:533–63. doi: 10.1007/s12028-023-01688-3, 36949360 PMC10241762

[7] Nolan JP Sandroni C Böttiger BW Cariou A Cronberg T Friberg H . European resuscitation council and European Society of Intensive Care Medicine guidelines 2021: post-resuscitation care. Resuscitation. (2021) 161:220–69. doi: 10.1016/j.resuscitation.2021.02.012, 33773827

[8] Snider SB Fischer D McKeown ME Cohen AL Schaper FLWVJ Amorim E . Regional distribution of brain injury after cardiac arrest: clinical and electrographic correlates. Neurology. (2022) 98:e1238–47. doi: 10.1212/WNL.0000000000013301, 35017304 PMC8967331

[9] Topjian AA Zhang B Xiao R Fung FW Berg RA Graham K . Multimodal monitoring including early EEG improves stratification of brain injury severity after pediatric cardiac arrest. Resuscitation. (2021) 167:282–8. doi: 10.1016/j.resuscitation.2021.06.020, 34237356 PMC8530861

[10] Kawai Y Kogeichi Y Yamamoto K Miyazaki K Asai H Fukushima H. Explainable artificial intelligence-based prediction of poor neurological outcome from head computed tomography in the immediate post-resuscitation phase. Sci Rep. (2023) 13:5759. doi: 10.1038/s41598-023-32899-5, 37031248 PMC10082754

[11] Chen C-C Massey SL Kirschen MP Yuan I Padiyath A Simpao AF . Electroencephalogram-based machine learning models to predict neurologic outcome after cardiac arrest: a systematic review. Resuscitation. (2024) 194:110049. doi: 10.1016/j.resuscitation.2023.110049, 37972682 PMC11023717

[12] Sun Y He Z Ren J Wu Y. Prediction model of in-hospital mortality in intensive care unit patients with cardiac arrest: a retrospective analysis of the MIMIC-IV database based on machine learning. BMC Anesthesiol. (2023) 23:178. doi: 10.1186/s12871-023-02138-5 PMC10210383 37231340

[13] Chen J Mei Z Wang Y Shen X Zeng R Chen Y . A nomogram to predict in-hospital mortality in post-cardiac arrest patients: a retrospective cohort study. Pol Arch Intern Med. (2023) 133:16325. doi: 10.20452/pamw.16325 35997470

[14] Rawshani A Hessulf F Deminger J Sultanian P Gupta V Lundgren P . Prediction of neurologic outcome after out-of-hospital cardiac arrest: an interpretable approach with machine learning. Resuscitation. (2024) 202:110359. doi: 10.1016/j.resuscitation.2024.110359, 39142467

[15] Lundberg SM Lee S-I. "A unified approach to interpreting model predictions". In: Guyon I, editor. Advances in Neural Information Processing Systems 30. Red Hook, NY, USA: Curran Associates (2017). p. 4765–74.

[16] Johnson AEW Bulgarelli L Shen L Gayles A Shammout A Horng S . MIMIC-IV, a freely accessible electronic health record dataset. Sci Data. (2023) 10:1. doi: 10.1038/s41597-022-01899-x, 36596836 PMC9810617

[17] Gaur S. K. P D. Abrol M. Assessing risk factors with generative adversarial networks for cardiac arrest detection 2024 International Conference on Optimization Computing and Wireless Communication (ICOCWC). (2024) 1–6

[18] Sahlaoui H Alaoui EAA Agoujil S Nayyar A. An empirical assessment of SMOTE variants techniques and interpretation methods in improving the accuracy and the interpretability of student performance models. Educ Inf Technol. (2024) 29:5447–83. doi: 10.1007/s10639-023-12007-w

[19] Raghuwanshi BS Shukla S. SMOTE based class-specific extreme learning machine for imbalanced learning. Knowl Based Syst. (2020) 187:104814. doi: 10.1016/j.knosys.2019.06.022

[20] Sachdeva RK Bathla P Rani P Solanki V Ahuja R. A systematic method for diagnosis of hepatitis disease using machine learning. Innov Syst Softw Eng. (2023) 19:71–80. doi: 10.1007/s11334-022-00509-8, 36628173 PMC9818056

[21] Jaya T. Mohan M. Alam M. S. Effective Heart Disease Prediction Using Machine Learning-Modified knn. Singapore: Springer Nature.

[22] Javeed A Saleem MA Dallora AL Ali L Berglund JS Anderberg P. Decision support system for predicting mortality in cardiac patients based on machine learning. Appl Sci (Basel). (2023) 13:5188. doi: 10.3390/app13085188

[23] Palanivinayagam A Damaševičius R. Effective handling of missing values in datasets for classification using machine learning methods. Information (Basel). (2023) 14:92. doi: 10.3390/info14020092

[24] Waseem MH Nadeem MSA Khan IR Shim SO Aziz W Habib U. Reinforcing artificial neural networks through traditional machine learning algorithms for robust classification of cancer. Comput Mater Contin. (2023) 75:4293–315. doi: 10.32604/cmc.2023.036710

[25] Yang S Cao L Zhou Y Hu C. A retrospective cohort study: predicting 90-day mortality for ICU trauma patients with a machine learning algorithm using XGBoost using MIMIC-III database. J Multidiscip Healthc. (2023) 16:2625–40. doi: 10.2147/JMDH.S416943, 37701177 PMC10493110

[26] Li Z. Extracting spatial effects from machine learning model using local interpretation method: an example of SHAP and XGBoost. Comput Environ Urban Syst. (2022) 96:101845–9715. doi: 10.1016/j.compenvurbsys.2022.101845, 42574925

[27] Seo DW Yi H Baek HJ Kim YJ Sohn CH Ahn S . Prediction of neurologically intact survival in cardiac arrest patients without pre-hospital return of spontaneous circulation: machine learning approach. J Clin Med. (2021) 10:1089. doi: 10.3390/jcm10051089 PMC7961400 33807882

[28] Ambale-Venkatesh B Yang X Wu CO Liu K Hundley WG McClelland R . Cardiovascular event prediction by machine learning: the multi-ethnic study of atherosclerosis. Circ Res. (2017) 121:1092–101. doi: 10.1161/CIRCRESAHA.117.311312, 28794054 PMC5640485

[29] Rajkomar A Oren E Chen K Dai AM Hajaj N Hardt M . Scalable and accurate deep learning with electronic health records. NPJ Dig Med. (2018) 1:18. doi: 10.1038/s41746-018-0029-1, 31304302 PMC6550175

[30] Kwon JM Jeon KH Kim HM Kim MJ Lim S Kim KH . Deep-learning-based out-of-hospital cardiac arrest prognostic system to predict clinical outcomes. Resuscitation (2019) 139:84–91. doi: 10.1016/j.resuscitation.2019.04.007 30978378

[31] Wormsbecker A Sekhon MS Griesdale DE Wiskar K Rush B. The association between anemia and neurological outcome in hypoxic ischemic brain injury after cardiac arrest. Resuscitation (2017) 112:11–6. doi: 10.1016/j.resuscitation.2016.12.010, 28007641

[32] SOS-KANTO study group. Relationship between the hemoglobin level at hospital arrival and post-cardiac arrest neurologic outcome. Am J Emerg Med. (2012) 30:770–4. doi: 10.1016/j.ajem.2011.03.031, 21641155

[33] Ameloot K Genbrugge C Meex I Janssens S Boer W Mullens W . Low hemoglobin levels are associated with lower cerebral saturations and poor outcome after cardiac arrest. Resuscitation. (2015) 96:280–6. doi: 10.1016/j.resuscitation.2015.08.015, 26325099

[34] Topjian AA Sutton RM Reeder RW Telford R Meert KL Yates AR . The association of immediate post cardiac arrest diastolic hypertension and survival following pediatric cardiac arrest. Resuscitation. (2019) 141:88–95. doi: 10.1016/j.resuscitation.2019.05.033, 31176666 PMC6709525

[35] Cardim D Griesdale DE Ainslie PN Robba C Calviello L Czosnyka M . A comparison of non-invasive versus invasive measures of intracranial pressure in hypoxic ischaemic brain injury after cardiac arrest. Resuscitation. (2019) 137:221–8. doi: 10.1016/j.resuscitation.2019.01.002, 30629992

[36] Annoni F Dell'Anna AM Franchi F Creteur J Scolletta S Vincent JL . The impact of diastolic blood pressure values on the neurological outcome of cardiac arrest patients. Resuscitation. (2018) 130:167–73. doi: 10.1016/j.resuscitation.2018.07.017, 30031784

[37] Hickey RW Painter MJ. Brain injury from cardiac arrest in children. Neurol Clin. (2006) 24:147–viii. doi: 10.1016/j.ncl.2005.10.002, 16443136

[38] Andrew E Nehme Z Bernard S Smith K. The relationship between age and outcome in out-of-hospital cardiac arrest patients. Resuscitation. (2015) 94:49–54. doi: 10.1016/j.resuscitation.2015.05.015 26044753

[39] Fink EL Berger RP Clark RS Watson RS Angus DC Richichi R . Serum biomarkers of brain injury to classify outcome after pediatric cardiac arrest*. Crit Care Med. (2014) 42:664–74. doi: 10.1097/01.ccm.0000435668.53188.80, 24164954 PMC4478619

[40] Rovlias A Kotsou S. The blood leukocyte count and its prognostic significance in severe head injury. Surg Neurol. (2001) 55:190–6. doi: 10.1016/S0090-3019(01)00414-1, 11358584

[41] Dufner MC Andre F Stiepak J Zelniker T Chorianopoulos E Preusch M . Therapeutic hypothermia impacts leukocyte kinetics after cardiac arrest. Cardiovasc Diagn Ther. (2016) 6:199–207. doi: 10.21037/cdt.2016.02.06, 27280083 PMC4880752

[42] Johnson AEW Kramer AA Clifford GD. A new severity of illness scale using a subset of acute physiology and chronic health evaluation data elements shows comparable predictive accuracy. Crit Care Med. (2013) 41:1711–8. doi: 10.1097/CCM.0b013e31828a24fe, 23660729

[43] Wang X Xia J Shan Y Yang Y Li Y Sun H. Predictive value of the Oxford acute severity of illness score in acute stroke patients with stroke-associated pneumonia. Front Neurol. (2023) 14:1251944. doi: 10.3389/fneur.2023.1251944, 37731859 PMC10507346

[44] Chen Q Zhang L Ge S He W Zeng M. Prognosis predictive value of the Oxford acute severity of illness score for sepsis: a retrospective cohort study. PeerJ. (2019) 7:e7083. doi: 10.7717/peerj.7083, 31218129 PMC6563807

[45] Grand J Lilja G Kjaergaard J Bro-Jeppesen J Friberg H Wanscher M . Arterial blood pressure during targeted temperature management after out-of-hospital cardiac arrest and association with brain injury and long-term cognitive function. Eur Heart J Acute Cardiovasc Care. (2020) 9:S122–30. doi: 10.1177/2048872619860804, 31246109

[46] Geocadin RG Koenig MA Jia X Stevens RD Peberdy MA. Management of brain injury after resuscitation from cardiac arrest. Neurol Clin. (2008) 26:487–506. doi: 10.1016/j.ncl.2008.03.015, 18514823 PMC3074242

[47] Irisawa T Matsuyama T Iwami T Yamada T Hayakawa K Yoshiya K . The effect of different target temperatures in targeted temperature management on neurologically favorable outcome after out-of-hospital cardiac arrest: a nationwide multicenter observational study in Japan (the JAAM-OHCA registry). Resuscitation (2018) 133:82–7. doi: 10.1016/j.resuscitation.2018.10.004 30316953

[48] Asmar S Chehab M Bible L Khurrum M Castanon L Ditillo M . The emergency department systolic blood pressure relationship after traumatic brain injury. J Surg Res. (2021) 257:493–500. doi: 10.1016/j.jss.2020.07.062, 32916502

[49] Brenner M Stein DM Hu PF Aarabi B Sheth K Scalea TM. Traditional systolic blood pressure targets underestimate hypotension-induced secondary brain injury. J Trauma Acute Care Surg. (2012) 72:1135–9. doi: 10.1097/TA.0b013e31824af90b, 22673237

[50] Fuller G Hasler RM Mealing N Lawrence T Woodford M Juni P . The association between admission systolic blood pressure and mortality in significant traumatic brain injury: a multi-Centre cohort study. Injury. (2014) 45:612–7. doi: 10.1016/j.injury.2013.09.008, 24206920

[51] Gul HF Dolanbay T Simsek AT Aras M. Evaluation of blood urea, creatinine, and glucose levels as biochemical indicators of the type and severity of traumatic brain injury. Turk Neurosurg. (2021) 31:333–8. doi: 10.5137/1019-5149.JTN.29843-20.2, 33372252

