# Supplementary material for: Early prediction of in-hospital acute brain injury following cardiac arrest using machine learning: development and external validation
Source: Front Neurol. 2026 Sep 18;17:1887511. doi: 10.3389/fneur.2026.1887511 (PMC13630667; doi:10.3389/fneur.2026.1887511)
Supplement: Supplementary file 1 [file Supplementary_file_1.DOCX]

**Supplementary Methods and Tables**

# **Supplementary Method S1. Missing-data and physiological-range handling**

Missingness was assessed separately for each candidate variable before model development. Variables with more than 50% missing observations were excluded; this criterion resulted in the exclusion of the Glasgow Coma Scale (GCS) total score and its eye, verbal, and motor components. The remaining variables were retained as candidate predictors.

The final datasets supplied to the modeling pipeline contained no blank predictor values. Nevertheless, all continuous predictors underwent an additional validity assessment using prespecified physiologically plausible ranges. Measurements outside these ranges were considered invalid and converted to missing values. The same validity criteria were applied to the development and external validation cohorts.

Physiological-range screening identified 91 invalid measurements in the MIMIC-IV development cohort and seven in the external validation cohort. Missing continuous values were imputed using medians estimated exclusively from the corresponding training data. During five-fold cross-validation, median values were estimated independently within each training fold and applied without refitting to the corresponding validation fold. For final evaluation, preprocessing parameters fitted using the complete MIMIC-IV training set were applied unchanged to the internal test and external validation cohorts. Neither outcome labels nor information from the internal test or external validation cohort was used to estimate the imputation parameters.

Gender, insurance status, mechanical ventilation, and acute kidney injury were treated as categorical or binary predictors and contained no missing values in the final modeling datasets. The nearest-value, linear-interpolation, and mean-interpolation approaches described in the original manuscript were not used in the revised analytical pipeline and have therefore been removed from the Methods.

## **Supplementary Table S1A. Candidate variables excluded because of missingness**

| **Variable** | **Unit** | **Development missing, n (%)** | **External missing, n (%)** | **Status** | **Reason** |
| --- | --- | --- | --- | --- | --- |
| GCS eye | points | 800 (56.377%) | Not collected | Excluded | >50% missing |
| GCS verbal | points | 912 (64.270%) | Not collected | Excluded | >50% missing |
| GCS motor | points | 834 (58.773) | Not collected | Excluded | >50% missing |
| GCS total | points | 912 (64.270%) | Not collected | Excluded | >50% missing |

## **Supplementary Table S1B. Physiological-range screening and imputation of included predictors**

| **Variable** | **Prespecified range** | **Development missing after screening, n (%)** | **External missing after screening, n (%)** | **Final handling** |
| --- | --- | --- | --- | --- |
| Age, years | 18-110 | 0 (0.000%) | 0 (0.000%) | Training median if missing |
| Gender | Binary | 0 (0.000%) | 0 (0.000%) | No imputation required |
| Weight, kg | 25-350 | 37 (2.607%) | 0 (0.000%) | Training median |
| Insurance | Binary | 0 (0.000%) | 0 (0.000%) | No imputation required |
| Heart rate, beats/min | 20-250 | 3 (0.211%) | 0 (0.000%) | Training median |
| Non-invasive systolic pressure, mmHg | 40-300 | 62 (4.369%) | 0 (0.000%) | Training median |
| Non-invasive diastolic pressure, mmHg | 20-200 | 62 (4.369%) | 1 (0.559%) | Training median |
| Non-invasive mean pressure, mmHg | 30-220 | 63 (4.440%) | 1 (0.559%) | Training median |
| Respiratory rate, breaths/min | 4-80 | 13 (0.916%) | 1 (0.559%) | Training median |
| SpO2, % | 50-100 | 6 (0.423%) | 1 (0.559%) | Training median |
| Temperature, degrees C | 30-43 | 49 (3.453%) | 3 (1.676%) | Training median |
| White blood cells, x10^9/L | 0.1-200 | 43 (3.030%) | 0 (0.000%) | Training median if missing |
| Red blood cells, x10^12/L | 0.5-8 | 44 (3.101%) | 0 (0.000%) | Training median if missing |
| Platelets, x10^9/L | 5-1,500 | 45 (3.171%) | 0 (0.000%) | Training median if missing |
| Hemoglobin, g/dL | 2-25 | 43 (3.030%) | 0 (0.000%) | Training median if missing |
| RDW, % | 5-40 | 44 (1.101%) | 0 (0.000%) | Training median if missing |
| Sodium, mmol/L | 100-180 | 40 (2.181%) | 0 (0.000%) | Training median if missing |
| Potassium, mmol/L | 1.5-10 | 41 (2.889%) | 0 (0.000%) | Training median if missing |
| Chloride, mmol/L | 60-150 | 40 (2,819%) | 0 (0.000%) | Training median if missing |
| Glucose, mg/dL | 20-1,500 | 41 (2.889%) | 0 (0.000%) | Training median |
| pH | 6.5-8.0 | 124 (8.739%) | 0 (0.000%) | Training median if missing |
| PCO2, mmHg | 5-200 | 140 (9.886%) | 0 (0.000%) | Training median if missing |
| Lactate, mmol/L | 0.1-30 | 136 (9.584%) | 0 (0.000%) | Training median if missing |
| Anion gap, mmol/L | 0-60 | 42 (2.960%) | 0 (0.000%) | Training median if missing |
| Total CO2, mmol/L | 2-80 | 140 (9.886%) | 0 (0.000%) | Training median if missing |
| Blood urea nitrogen, mg/dL | 1-300 | 39 (2.748%) | 0 (0.000%) | Training median if missing |
| Creatinine, mg/dL | 0.1-30 | 40 (2.819%) | 0 (0.000%) | Training median if missing |
| Magnesium, mg/dL | 0.2-10 | 44 (3.101%) | 0 (0.000%) | Training median if missing |
| SOFA | 0-24 | 0 (0.000%) | 0 (0.000%) | Training median if missing |
| SIRS | 0-4 | 0 (0.000%) | 0 (0.000%) | Training median if missing |
| SAPS II | 0-163 | 0 (0.000%) | 0 (0.000%) | Training median if missing |
| OASIS | 0-75 | 0 (0.000%) | 0 (0.000%) | Training median if missing |
| Mechanical ventilation | Binary | 0 (0.000%) | 0 (0.000%) | No imputation required |
| Acute kidney injury | Binary | 0 (0.000%) | 0 (0.000%) | No imputation required |

*Values outside the prespecified physiological ranges were converted to missing before model fitting. Percentages use N=1,419 for development and N=179 for external validation. Imputation parameters were estimated exclusively from the corresponding training data.*

# **Supplementary Method S2. External validation cohort and variable harmonization**

The external validation cohort comprised 179 patients with in-hospital cardiac arrest treated at Tianjin Medical University General Hospital between 2022 and 2024. The cohort included 27 patients with newly documented acute brain injury and 152 without acute brain injury. Eligibility criteria, predictor definitions, measurement units, and temporal windows were harmonized with those used for the MIMIC-IV cohort.

Predictors were collected during the first 24 hours after ICU admission. Patients with acute brain injury documented before or during this predictor window were excluded, and the outcome was assessed from the end of the 24-hour window until hospital discharge.

The hospital dataset originally contained no blank values for the model predictors. Physiological-range screening identified seven invalid measurements, which were converted to missing and imputed using medians estimated exclusively from the MIMIC-IV training data. The external cohort was not used for feature selection, hyperparameter optimization, oversampling, threshold selection, imputation fitting, or model training.

## **Supplementary Table S2. Comparison of the development and external validation cohorts**

| **Characteristic** | **MIMIC-IV cohort (N=1,419)** | **External cohort (N=179)** | **Absolute SMD** |
| --- | --- | --- | --- |
| PCABI, n (%) | 250 (17.6%) | 27 (15.1%) | 0.068 |
| Age, years | 68.00 (57.00-81.00) | 67.00 (56.00-81.00) | 0.023 |
| Male sex, n (%) | 842 (59.3%) | 108 (60.3%) | 0.020 |
| Hemoglobin, g/dL | 11.50 (9.80-13.30) | 11.70 (9.65-13.70) | 0.005 |
| OASIS | 39.00 (32.00-47.00) | 42.00 (32.00-47.00) | 0.107 |
| Temperature, degrees C | 36.61 (36.17-37.00) | 36.72 (36.20-37.09) | 0.172 |
| BUN, mg/dL | 23.00 (16.00-37.00) | 22.00 (16.00-32.50) | 0.101 |
| White blood cells, x10^9/L | 11.90 (8.30-16.80) | 11.72 (8.20-16.30) | 0.046 |
| Non-invasive systolic pressure, mmHg | 121.00 (103.00-138.00) | 125.00 (109.09-140.00) | 0.021 |
| Non-invasive diastolic pressure, mmHg | 68.00 (56.00-82.00) | 69.00 (57.00-84.00) | 0.002 |
| Mechanical ventilation, n (%) | 1,258 (88.7%) | 164 (91.6%) | 0.100 |
| Predictor window | First 24 h | First 24 h | - |
| Outcome window | >24 h to discharge | >24 h to discharge | - |
| Brain injury during predictor window excluded | Yes | Yes | - |

*Continuous variables are median (interquartile range); categorical variables are n (%). SMD, standardized mean difference; PCABI, post-cardiac-arrest brain injury.*

#### **Supplementary Method S3. Calibration Assessment**

Calibration of the final RF model was evaluated separately in the internal test and external validation cohorts. Calibration plots were constructed by comparing predicted probabilities with the observed proportions of subsequent in-hospital acute brain injury across the range of predicted risk. The 45-degree reference line represented perfect agreement between predicted and observed risks.

Calibration was additionally summarized using the Brier score, calibration intercept, and calibration slope. The Brier score quantified the overall difference between predicted probabilities and observed outcomes, with lower values indicating better probabilistic accuracy. An intercept of 0 and a slope of 1 represented ideal calibration. All measures were calculated from predictions generated by the final RF model. Preprocessing parameters and model settings derived from the development cohort were applied unchanged to the internal test and external validation cohorts, without refitting or recalibration.

**Supplementary Figure S1.** Calibration of the random forest model in the internal test and external validation cohorts. The calibration curves compare predicted probabilities with the observed proportions of subsequent in-hospital acute brain injury. The dashed diagonal line represents perfect agreement between predicted and observed risks. Predictions were generated using the final model without refitting or recalibration in either cohort.
